# Abnormal Human Chondrocyte Morphology is Related to Increased Levels of Cell-Associated IL-1β and Disruption to Pericellular Collagen Type VI

**DOI:** 10.1002/jor.21155

**Published:** 2010-04-24

**Authors:** Dianne H Murray, Peter G Bush, Ivan J Brenkel, Andrew C Hall

**Affiliations:** 1 Centre for Integrative Physiology, School of Biomedical Sciences, University of EdinburghHugh Robson Building, George Square, Edinburgh EH8 9XD, Scotland, United Kingdom; 2 Department of Orthopaedics and Trauma, Queen Margaret Hospital, Fife Acute Hospitals NHS TrustDunfermline, Fife KY12 0SU, Scotland, United Kingdom

**Keywords:** osteoarthritis, chondrocyte, collagen type VI, interleukin, morphology

## Abstract

Early osteoarthritis (OA) is poorly understood, but abnormal chondrocyte morphology might be important. We studied IL-1β and pericellular collagen type VI in morphologically normal and abnormal chondrocytes. In situ chondrocytes within explants from nondegenerate (grade 0/1) areas of human tibial plateaus (*n* = 21) were fluorescently labeled and visualized [2-photon laser scanning microscopy (2PLSM)]. Normal chondrocytes exhibited a “smooth” membrane surface, whereas abnormal cells were defined as demonstrating ≥1 cytoplasmic process. Abnormal chondrocytes were further classified by number and average length of cytoplasmic processes/cell. IL-1β or collagen type VI associated with single chondrocytes were visualized by fluorescence immuno-histochemistry and confocal laser scanning microscopy (CLSM). Fluorescence was quantified as the number of positive voxels (i.e., 3D pixels with fluorescence above baseline)/cell. IL-1β-associated fluorescence increased between normal and all abnormal cells in the superficial (99.7 ± 29.8 [11 (72)] vs. 784 ± 382 [15 (132)]; *p* = 0.04, positive voxels/cell) and deep zones (66.5 ± 29.4 [9 (64)] vs. 795 ± 224 [9 (56)]; *p* = 0.006). There was a correlation (*r*^2^ = 0.988) between the number of processes/cell (0–5) and IL-1β, and an increase particularly with short processes (≤5 µm; *p* = 0.022). Collagen type VI coverage and thickness decreased (*p* < 0.001 and *p* = 0.005, respectively) with development of processes. Abnormal chondrocytes in macroscopically nondegenerate cartilage demonstrated a marked increase in IL-1β and loss of pericellular type VI collagen, changes that could lead to cartilage degeneration. © 2010 Orthopaedic Research Society. Published by Wiley Periodicals, Inc. J Orthop Res 28:1507–1514, 2010

Osteoarthritis (OA) is a complex disorder involving articular cartilage degeneration of diarthrodial joints^1^ in which changes to matrix metabolism by chondrocytes are pivotal.^2^,^3^ Alterations to the balance between matrix synthesis/breakdown occur,^2^,^3^ but as degeneration is slow, early changes are difficult to identify. However, the study of chondrocytes in nondegenerate cartilage could be productive.^4^ Using single and 2-photon laser scanning microscopy (2PLSM),^5^,^6^ we have shown that the morphology of living in situ chondrocytes in relatively nondegenerate (grade 0/1) tibial plateau cartilage is complex. The elliptical shape of superficial zone (SZ) cells was evident, in addition to rounded forms in mid and deep zones (MZ, DZ). However, a proportion (∼40%) of cells was abnormal with cytoplasmic processes^5^ an observation confirmed by others also reporting abnormal human chondrocytes.^7^–^9^ These processes differ from the primary cilia observed in articular chondrocytes as the latter are shorter (∼1 µm) requiring specific cytoskeletal labeling.^10^

Chondrocytes are phenotypically unstable^11^ and demonstrate a close relationship between shape and matrix metabolism. For example, chondrocytes in 3D culture synthesize cartilage-specific macromolecules (type II collagen, aggrecan^12^,^13^) but if the cell–matrix interaction is modified (e.g., in 2D culture), changes to a fibroblastic phenotype occur^12^ with alterations to matrix metabolism (increased collagen type I and small proteoglycan production).^12^–^14^ In 3D cultures, de-differentiated cells revert to the chondrocytic phenotype with recovery in synthesis of cartilage-specific macromolecules.^14^ Thus chondrocytes with abnormal morphology could be associated with deleterious changes to matrix metabolism, potentially leading to OA.

ECM breakdown in OA is largely mediated by raised levels of pro-inflammatory cytokines (e.g., IL-1β and TNF-α) and mediators, which stimulate the synthesis/release of degradative enzymes^2^,^3^ and inhibit aggrecan/collagen synthesis.^15^,^16^ IL-1β is highly expressed by chondrocytes and in synovial fluid of OA joints^17^,^18^ and it induces cartilage damage, for example, by elevating matrix metalloproteinases (MMPs).^2^,^3^,^19^,^20^ Changes to levels of cytokines/degradative enzymes alter the pericellular matrix (PCM; “chondron”^21^) around chondrocytes. This can be determined by studying collagen type VI which is exclusively localized to the PCM in nondegenerate cartilage^22^–^24^ and defines the extent/integrity of the chondron.^22^ Accumulation of collagen type VI around human chondrocytes is suppressed by IL-1β^25^ and disrupted in OA.^24^–^26^

We investigated whether abnormal chondrocyte morphology in relatively nondegenerate cartilage^5^ was related to increased levels of cell-associated IL-1β. As IL-1β is linked to matrix breakdown,^15^,^16^ we also measured collagen type VI distribution to assess the PCM of abnormal chondrocytes. We incubated osteochondral explants of human tibial plateau cartilage graded 0 or 1^5^ with a vital fluorescent dye to label in situ cells. Using CLSM/2PLSM and image analysis,^5^,^6^,^27^ we recorded fine cell shape to discriminate between normal (elliptical/rounded) or abnormally shaped chondrocytes (i.e., with ≥1 cytoplasmic processes/cell). Using fluorescence immuno-histochemistry (FI) on the same cells, we visualized IL-1β labeling and PCM collagen type VI. FI was quantified to statistically compare labeling between cells of varying morphology. IL-1β levels were markedly elevated in abnormal cells, with collagen type VI coverage/thickness reduced compared to normal cells. This suggests that abnormal chondrocytes in relatively nondegenerate cartilage are associated with marked changes to levels of a cytokine implicated in OA and their PCM was compromised.

## MATERIALS AND METHODS

### Biochemicals

CellTracker™ Blue (CTB) was from Invitrogen (Paisley, UK). Optimal cutting temperature compound (OCTC) was from R. Lamb Ltd (Eastbourne, UK). Rabbit monoclonal anti-IL-1β (1:200 in PBS) and rabbit polyclonal anti-collagen type VI (1:50) were from Abcam (Cambridge, UK). Fluorescently tagged secondary antibody (2°Ab) goat anti-rabbit IgG Alexa Fluor 546 (1:50) was from Invitrogen. Fluosave was from Calbiochem (Beeston, UK) and other biochemicals from Sigma–Aldrich (Poole, UK).

### Human Cartilage

Tissue was obtained with ethical permission from the tibial plateau of 21 patients (13 males and 8 females; range 49–86 years; 68.4 ± 2.3 years) undergoing total knee arthroplasty. Excised joints showed various stages of OA and were graded as described.^5^ Only tibial plateau cartilage with intact SZ (grades 0/1) and underlying bone attached was used. Tissue blocks were trimmed to ∼2 mm × 5 mm × 5 mm,^28^ then incubated in DMEM with CTB (5 µM; 2 h; 37°C) to fluorescently label live in situ chondrocytes. Samples were snap-frozen in freezing hexane (−80°C), sections cut (25 µm; −25°C) using OCTC, mounted on silane-coated slides, and stored (−80°C) until required.

### Fluorescence Immuno-Histochemistry

**Figure 4:**
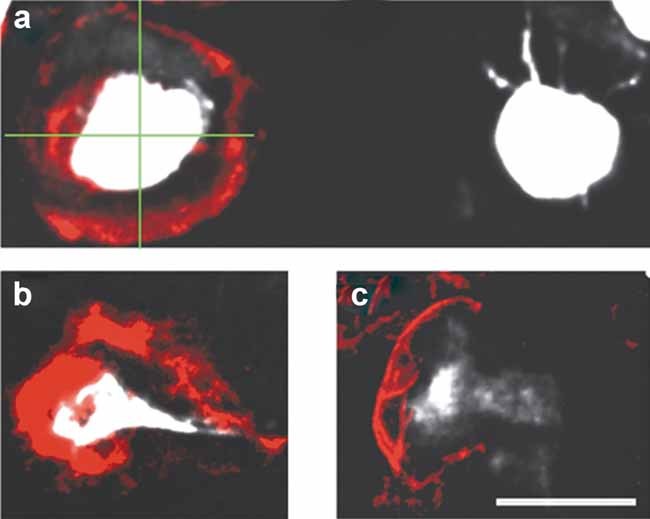
Examples and assessment of collagen type VI labeling-associated fluorescence with in situ chondrocytes. Collagen around single chondrocytes was analyzed by overlaying vertical/ horizontal lines (green overlay, (a)), on projected images meeting at the cell center. The number of line intersections (0–4) with collagen staining was recorded as a measure of encapsulation. At each intersection, the thickness of collagen staining was measured perpendicular to the stained edge. Labeling is shown completely (∼100%) enclosing a normal cell (a), or absent from an adjacent abnormal cell (∼0%), or surrounding (b) ∼75%, or (c) ∼50% of abnormal cells (bar = 10 µm).

For IL-1β, sections were air-dried before antigen retrieval^8^ then washed (PBS; 3 × 5 min) and permeabilized (Triton X-100 0.5%; 5 min; 21°C). After 6× further washes, nonspecific 2°Ab binding was blocked with serum from the 2°Ab production host species. For collagen type VI, antigen retrieval was not required and nonspecific 2°Ab binding was blocked as above. Staining was performed with the 1°Ab (2 h; 37°C), washed and incubated with the 2°Ab (1 h; 37°C). After a final wash, samples were mounted (Fluosave) and stored (4°C) until imaged. Controls were treated as above except for the 1°Ab, which was replaced with serum from the 1°Ab production host species and these showed no detectable fluorescence (data not shown). The best 1°Abs trialed for both IL-1β and the collagen were from the same host species making dual FI impossible. Controls showed specific IL-1β labeling to normal human synovial cells was undetectable, but markedly increased in inflamed tissue (data not shown). Collagen type VI labeling was specific to the PCM^22^–^26^ (data not shown; see Fig. 4).

### CLSM/2PLSM Image Acquisition and Analysis of Fluorescently Labeled Proteins

**Figure 1:**
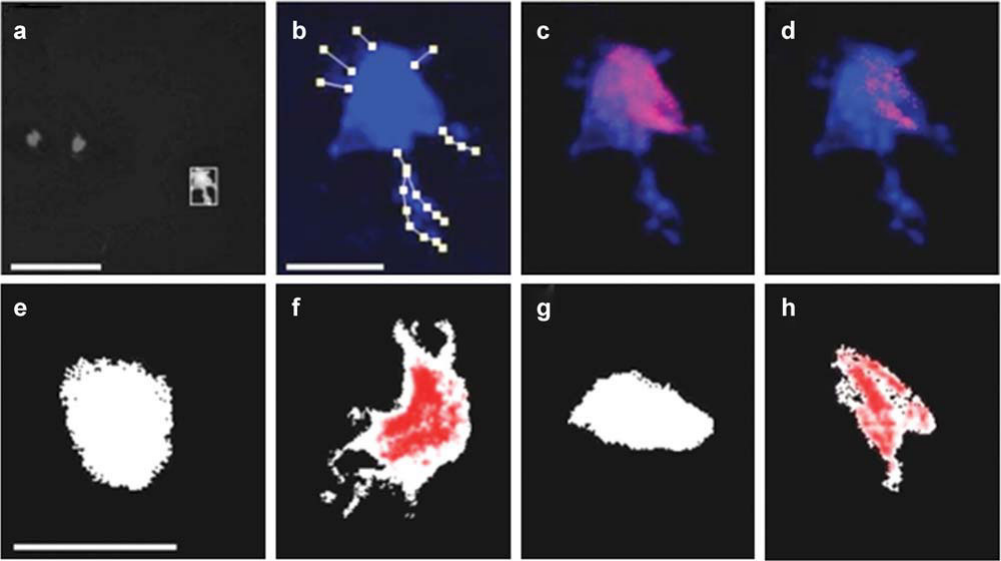
Cell morphology and quantification of cell-associated IL-1β fluorescence. Image (a) shows a cell labeled with CellTracker™ Blue (CTB; shown white for identification) selected from a field of view taken at low magnification (bar = 50 µm). This cell is shown at higher magnification (image (b); bar = 10 µm) with morphology (number and average length of cytoplasmic processes) of the CTB-labeled chondrocyte determined by lines drawn along processes in a 3D projected image measured by Volocity™. The cell was then isolated as a region of interest (ROI) by selecting a point of intense immuno-fluorescence within the cell's center (image (c)) and adjacent voxels included as part of the cell if within 40% of the CTB signal with the ROI shown with associated immuno-fluorescence. IL-1β immuno-fluorescence was subjected to an intensity threshold (image (d)) determined from in situ chondrocytes on control slides and the remaining positive voxels reported using Volocity™. Examples of IL-1β fluorescence immuno-histochemistry in cells with varying morphology are shown (images (e–h); bar for all = 10 µm). CTB fluorescence was shown white with IL-1β immuno-fluorescent positive voxels overlaid in red. In image (e), a normal SZ chondrocyte is shown, with negligible levels of cell-associated IL-1β immuno-fluorescence, whereas the SZ cell shown in (f) was abnormal and demonstrated marked IL-1β labeling. A normal DZ chondrocyte is also shown with no detectable IL-1β labeling (image (g)), and image (h) illustrates an abnormal DZ chondrocyte with a cytoplasmic process and clear IL-1β labeling.

A Zeiss Axioskop LSM510 (Welwyn Garden City, UK) with Verdi/Mira pulsed laser (Coherent, Inc., Santa Clara, CA) and 63× water immersion (NA = 1.2) or 10× dry (NA = 0.3) objectives were used to obtain images of CTB (cell morphology^5^,^6^) and Alexa Fluor 546 (IL-1β/collagen type VI-associated fluorescence). For CTB, Ex = 780 nm (2-P excitation^6^), Em = 435–485 nm; for Alexa Fluor, Ex = 543 nm, Em = 560 nm. *z*-Axis increments were typically 1 µm and image quality optimized.^5^,^6^ Morphology was recorded in 3D (Volocity™, Perkin Elmer, Cambridge, UK). Cytoplasmic processes yielded low fluorescence compared to the cell body at low magnification (Fig. 1a); however, at high power, fine processes were easily identified (Fig. 1b). We inspected each optical section, then traced, and measured their length and number in 3D of single cells by eye. Chondrocytes with “normal” morphology exhibited a “smooth” membrane surface, whereas “abnormal” cells were defined as demonstrating ≥1 cytoplasmic process. Abnormal chondrocytes were further classified by the number and average length of cytoplasmic processes per cell. Usually only cells in the center of a section, with processes <8 µm from the cell body in the *z*-axis could be analyzed precisely. For longer processes, accurate lengths were obtained in *x* and *y*. Our protocols under, rather than over-estimated the number/length of processes for cells at the edges of sections. Fluorescent IL-1β labeling was determined on a per cell basis using Volocity™. Unbiased selection of cells was performed by taking a point of intense CTB fluorescence within the cell's center, with adjacent voxels (3D pixels) selected if within ∼40% of the point intensity. Voxels of anti-IL-1β-associated fluorescence were only counted if co-localized with CTB and above threshold (Fig. 1c,d), which was established from the maximum voxel intensity of AlexaFluor for all cells in the negative controls, imaged using identical parameters to the positively stained sections. Thus, only IL-1β-associated fluorescence co-localizing with CTB (and thus chondrocyte-associated) was used in our analysis. For collagen type VI, single CTB-labeled cells were identified, and Ab staining measured for thickness/degree of coverage. A cross hair was placed over the cell and where the lines intersected the fluorescence, thickness was measured and averaged (Fig. 4a). The number of intersections (0–4) identified the degree of encapsulation (0–100%).

### Statistics

The study was performed on 21 subjects who contributed one joint each (i.e., *n* = 21). *N* represented the total number of chondrocytes studied at each condition and data are presented as mean ± SEM for [*n* (*N*)]. Statistical tests were performed using Sigma Stat (Systat, Inc., San Jose, CA). Unpaired Student's *t*-test and Mann–Whitney rank sum test (indicated with a single asterisk, *) were used to compare normally and nonnormally distributed data, respectively. Trends were analyzed by least-squares regression and Kruskal–Wallis one-way ANOVA. Resulting *p*-values are indicated and considered significant at *p* < 0.05.

## RESULTS

### Sample Population and Cartilage Quality

Many tibial plateaus were screened with only 21 joints having sufficiently large areas on gross inspection to be nondegenerate. After microscopic examination of the surface, only the cartilage from areas of two joints was grade 0, with the rest being grade 1, that is, some surface roughness but no loss of SZ chondrocytes.^5^ Thus, grade 0 and 1 cartilages were considered nondegenerate and the data pooled.

### Morphology of In Situ Human Chondrocytes

Chondrocyte heterogeneity can only be fully appreciated using fluorescent labeling and CLSM/2PLSM.^5^,^6^,^27^ The number (1–9) and length (∼1 to ∼40 µm) of the processes varied markedly. Morphology was classified as either normal (elliptical/spheroidal) with a “smooth” surface, or abnormal, that is, a chondrocyte with one or more cytoplasmic processes. Of the 677 cells examined, 311 (∼46%) exhibited normal rounded morphology; however, we actively sought out morphologically abnormal cells so that the full range of morphology could be represented and the relationship between shape and cell-associated IL-1β and collagen VI levels determined. Abnormal cells were defined as having one or more cytoplasmic processes. These cells were further classified on the basis of the number/average length of processes per cell. The groups for the number of processes/cell ranged from none (P_0_; normal morphology), one (P_1_), two (P_2_), three (P_3_), four (P_4_), and five (P_5_). Cells with P_≥6_ were observed, but not in a sufficient number of independent joints for evaluation. Classification was also based on the average length of the cytoplasmic processes/cell, and grouped as; L_0_ (normal morphology), L_5_ (≤5 µm), L_10_ (5–10 µm), L_15_ (10–15 µm), and L_>15_ (>15 µm). This classification underestimated the variety of cell shapes present; however they were appropriate groupings for this study.

### Chondrocyte Morphology and Cell-Associated IL-1β

**Figure 2:**
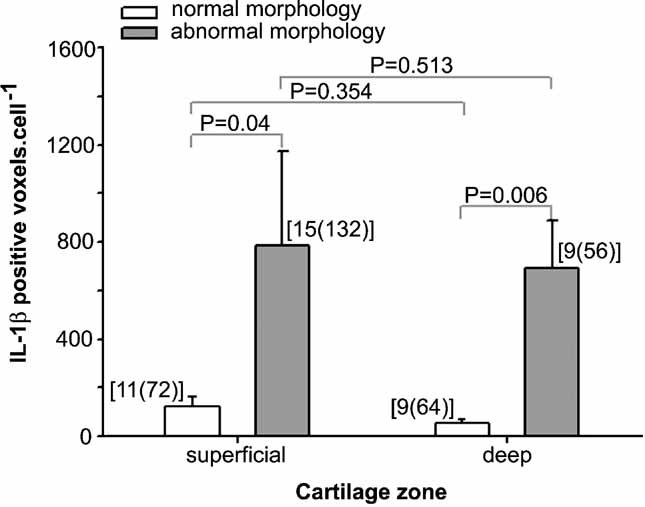
IL-1β immuno-fluorescence associated with normal or abnormal cells in the superficial zone (SZ) or deep zone (DZ). There was significantly greater IL-1β fluorescence in morphologically abnormal chondrocytes (i.e., cells with one or more cytoplasmic process) compared to normal cells in both the SZ and DZ. There was no difference between normal or abnormal cells in the two zones (*p* = 0.354, 0.513). Data as means ± SEM for [*n* (*N*)] and compared using unpaired Student's *t*-tests.

**Figure 3:**
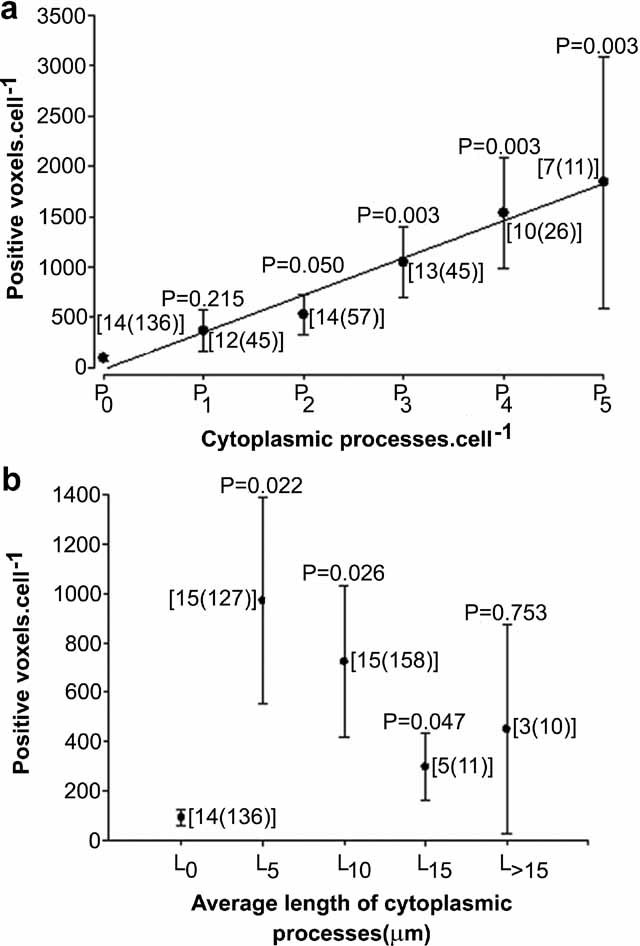
IL-1β immuno-fluorescence as a function of chondrocyte morphology. Cell morphology was categorized by (a) the number of processes/cell and levels of cell-associated IL-1β fluorescence determined. IL-1β labeling increased with the number of processes (linear regression; *r*^2^ = 0.988). IL-1β immuno-fluorescence was significantly higher compared to control (P_0_) for P_2_–P_5_ cells. Morphology was also categorized by (b) the average length of processes/cell and IL-1β levels were significantly higher for cells up to L_15_ compared to normal (L_0_), but not for L_>15_ cells (*p* = 0.753). Data were compared using unpaired Student's *t*-tests.

Figure 1e–h shows examples of normal and abnormal cells in the SZ and DZ with IL-1β levels identified by FI. By counting the number of positively stained voxels (i.e., 3D pixels containing fluorescence above baseline), we statistically compared cell-associated IL-1β fluorescence for cells of different morphology. MZ chondrocytes were not analyzed as they were difficult to identify as there was often not a clear demarcation between zones.^29^ IL-1β labeling increased for abnormal cells in both SZ and DZ (Fig. 2; *p* = 0.04 and 0.006) whereas there was no difference between normal (*p* = 0.354) or abnormal cells (*p* = 0.513) in the two zones suggesting abnormal morphology determined IL-1β levels rather than the zone in which the chondrocyte resided. When the numbers of processes/cell were compared, there was a significant (∼270+ve voxels/cell process) linear correlation (Fig. 3a). Cells in groups P_2_–P_5_ had more positive voxels than normal cells (*p* = 0.05 for P_2_; *p* = 0.003 for P_3_, P_4_, and P_5_). When IL-1β FI was compared between cells grouped by average process length (Fig. 3b), there was an increase for L_5_, L_10_, and L_15_ (*p* = 0.022, 0.026, 0.047). However, IL-1β levels decreased with average length of processes from L_5_ to L_15_. For L_>15_ cells, there was no difference compared to normal (*p* = 0.753) although the number of joints and cells in this group was small and the error large. Thus abnormal chondrocytes, particularly those with ≥2 processes/cell, and those where the average length of the processes was ≤5 µm had higher levels of cell-associated IL-1β labeling compared to normal cells.

### Collagen Type VI and Chondrocyte Morphology

**Figure 5:**
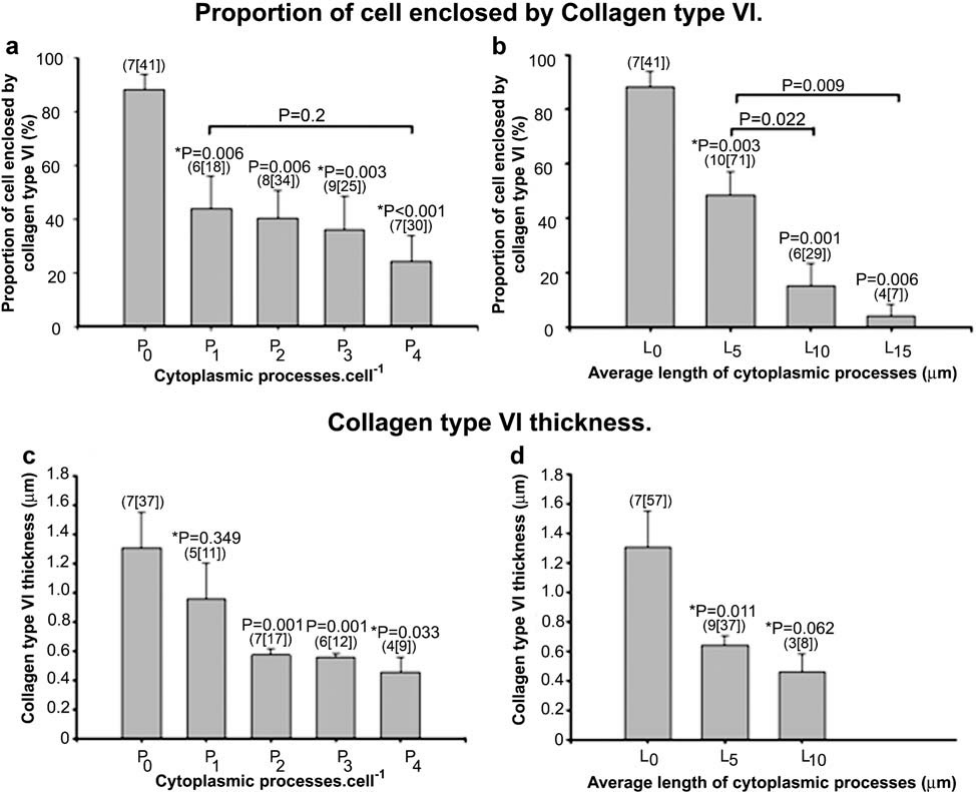
Collagen type VI associated with SZ chondrocytes of varying morphology. The % of collagen type VI around cells is shown in relation to (a) the number of processes/cell or (b) the average length of processes/cell. For P_1–4_ there was a reduction in % encapsulation with number of processes/cell compared to P_0_ cells (*p* = 0.006 or less) but no difference between groups of increasingly abnormal cells (*p* = 0.2; ANOVA). Compared to L_0_ cells (b), there was a decrease for all groups (*p* = 0.003 or less) and L_10_ and L_15_ cells exhibited a reduction compared to L_5_ cells (*p* = 0.022 and 0.009, respectively). The lower panels show collagen thickness around normal/abnormal chondrocytes (number of processes/cell, c) or average length of processes/cell (d). Collagen thickness (c) was reduced for P_2–4_ compared to P_0_ cells. L_5_ cells showed a reduction (*p* = 0.011) compared to L_0_ but the decrease for L_10_ was not significant (*p* = 0.062). Data were shown as means ± SEM for [*n* (*N*)] and compared by unpaired Student's *t*-tests, or Mann–Whitney rank sum tests (indicated by ^*^).

SZ cells were studied as the full range of shapes was present, and labeling performed in parallel with assessment of chondrocyte shape. Figure 4a shows a normal cell beside an abnormal chondrocyte, with very different collagen type VI levels (∼100% vs. ∼0%) as the normal cell was surrounded whereas the abnormal cell was devoid of this collagen. The method for assessing collagen type VI encapsulation and thickness around in situ chondrocytes is also shown with examples of a cell with (b) ∼75% and (c) ∼50% encapsulation. Collagen type VI almost completely (88.4 ± 5.53%; Fig. 5a,b) surrounded normal cells. There was a reduction in type VI enclosure when morphology was classified by the number of processes (Fig. 5a). P_1_ cells had ∼50% of the type VI collagen of P_0_ cells (*p* = 0.006); however, for cells with >1 process, there was no further decrease (*p* = 0.2). There was a large reduction in collagen coverage as the average process length increased (L_5_–L_10_ and L_15_; *p* < 0.001 by ANOVA; Fig. 5b). Collagen type VI thickness declined with number of cytoplasmic processes/cell and reached a lower level (P_2_, P_3_, and P_4_; *p* = 0.001, 0.001, and 0.033; Fig. 5c) compared to normal cells. Abnormal cells showed reduced (*p* = 0.011) collagen thickness with average process length for L_5_, but the decrease for L_10_ cells was not quite significant (*p* = 0.062; Fig. 5d).

## DISCUSSION

This study extends previous work^5^,^15^,^16^ by showing that abnormal morphology of in situ human chondrocytes in relatively nondegenerate load-bearing articular cartilage was associated with increased IL-1β levels particularly in chondrocytes with many processes/cell (Fig. 3a) and when the processes were short (Fig. 3b). Our relatively novel approach for FI quantification permitted a statistical analysis of cell-associated Ab labeling. Collagen type VI coverage was reduced in abnormal chondrocytes especially those with a large average length of processes (Fig. 5). The results suggested that changes to human chondrocyte morphology occur in relatively nondegenerate cartilage with marked effects on cell-associated levels of IL-1β and collagen type VI of the PCM.

We used aged and relatively nondegenerate cartilage, which might not be exactly comparable to “normal” tissue as it was taken from discrete areas of failed joints. Care was taken to ensure only load-bearing grade 0/1 areas were studied. Obtaining grade 0 human cartilage can be difficult; however, since OA is focal with adjacent nondegenerate areas, we used the present approach.^4^ We only had access to two grade 0 samples, and for some of the others it was difficult to determine if they were grade 0 or 1. Thus, we pooled this tissue, so the crucial comparison was between the range of cell shapes rather than cartilage grades. It might be thought there would be an inverse relationship between % normal chondrocytes and patient age. Although the age range (49–86 years) and patient number (21) was limited, there was no change (*r*^2^ = −0.088 ± 0.141% normal cells/year; slope *N*/*S*; *p* = 0.5). In addition, IL-1β levels increased in abnormal cells (Figs. 2 and 3), and this cytokine induces changes to matrix metabolism in complete contrast to those occurring in aged cartilage.^30^

Although we detected very low levels of cell-associated IL-1β in normal chondrocytes (∼100 voxels/cell; Figs. 2 and 3) we regard this as a negligible/baseline level. Of all the 320 cells studied (Fig. 3a) only 25% had significant IL-1β labeling, but this was not a quarter of all cells in cartilage. These highly abnormal cells (≥3 processes) were only a small % of the population and almost all were in the SZ, where IL-1β levels were highest.^16^ We actively sought out abnormal cells and thus this does not represent the whole cell population. Furthermore, the % of abnormal cells in the DZ of grade 0/1 cartilage was low (typically ∼5%) compared to the SZ; however, IL-1β levels were elevated to the same extent as abnormal cells in the SZ (Fig. 2). Thus, *cell shape* and not *cartilage zone* determined IL-1β levels. An important question is whether the IL-1β of abnormal chondrocytes was locally expressed in an auto-/paracrine manner, or whether it primarily originated from synovial fluid. Levels of IL-1β in synovial fluid rise markedly during OA,^17^ and this, with the increased permeability of a weakened matrix, could increase levels in the SZ and around chondrocytes. This cytokine could stimulate IL-1β expression and this taken with the higher levels of the cytokine receptor (IL-1R1) in chondrocytes from OA patients^31^ raises the possibility that abnormal chondrocytes bind more IL-1β. It is possible that both sources of IL-1β could be involved although their relative contributions are unclear. However, in preliminary experiments (A.C. Hall, P.G. Bush, unpublished work) where we have incubated cartilage explants for up to 2 weeks with high levels of IL-1β (50 ng/ml) compared to those found in synovial fluid of OA patients,^17^ we have not observed abnormal chondrocyte morphology. Thus, although more work is clearly required, we think it is unlikely that the abnormal chondrocytes observed in the present study arise from normal chondrocytes exposed to exogenous IL-1β.

Irrespective of the origin of the IL-1β, this cytokine could stimulate autocrine response(s) and signaling cascades leading to local release/activation of matrix-degrading enzymes (MMPs, ADAMTS^2^,^19^,^20^,^32^–^34^) causing a weakened matrix through which processes develop, similar to the proteolysis of cell invasion through the ECM.^35^ Alternatively, abnormal cells could result from mechanical damage to collagen type VI-PCM integrity^23^ leading to reduced cell–matrix interactions.^23^,^36^ Loss of this collagen could enhance chondrocyte migration through gaps in the chondron capsule.^37^ A close relationship exists between cell shape, expression of matrix constituents and degradative enzymes.^11^–^14^ However, the role of IL-1β is unclear as it could also be involved in matrix anabolism/repair.^38^ The association between abnormal chondrocytes, fibroblastic phenotype, and matrix turnover^11^–^14^ is important. However, techniques for visualizing/categorizing in situ chondrocyte shape and correlating this with quantitative levels of fluorescently labeled proteins are not trivial requiring further development, with concerns including the CLSM system/imaging software, methods for quantification, Ab specificity, and antigen retrieval.

Reduced collagen type VI thickness/coverage (Fig. 5) could be important for abnormal chondrocyte development in macroscopically nondegenerate cartilage. Interestingly, in the collagen type VI knockout mouse, the PCM develops normally but has reduced stiffness *prior to* the onset of histological changes associated with OA.^39^ Although changes to cell–matrix interactions probably occur, early events relating changes to PCM, cell shape, and IL-1β levels are unknown. Our data suggest that levels of cell-associated IL-1β are higher in abnormal cells when the average length of the processes was ≤5 µm (Fig. 3b) and these cells had reduced collagen type VI encapsulation (Fig. 5b). Early PCM weakening could lead to a loss of chondrocyte–matrix interactions and development of multiple short processes, raising levels of cell-associated IL-1β compared to when the average length of processes is longer (Fig. 3b). Increased cytokine levels would stimulate the release of degradative enzymes^2^ leading to further PCM breakdown and focal areas of weakness around chondrocytes through which the development of a small number of larger cytoplasmic processes develops. Once the processes break out of the PCM, then the ability of the chondrocyte to maintain the PCM decreases leading to increased loss of collagen type VI (Fig. 5b). With our methods we cannot determine if the chondrocytes with cytoplasmic processes are on the point of dying but it is possible these cells are more sensitive to mechanical loading compared to normal rounded/ellipsoidal chondrocytes.

Caution should however be applied when comparing our collagen type VI data to previous work, as we used grade 0/1 cartilage and labeling was related to cell morphology in contrast to the generalized changes in severely degenerate cartilage (grade 2–3) with chondrocyte clusters.^24^–^26^ These studies showed increased type VI collagen synthesis by cells in the lower MZ/upper DZ, whereas there was a loss in the upper MZ^24^,^26^ possibly related to PCM remodeling with chondrocyte proliferation/clustering in late OA.^24^–^26^ A previous study^26^ noted viable “exceptional” chondrocytes with low collagen type VI, and it is possible that these were abnormally shaped cells (Fig. 5). In summary, abnormal in situ chondrocyte morphology in relatively nondegenerate human cartilage was associated with increased cell-associated IL-1β levels and loss of pericellular collagen type VI structure. A future challenge will be to clarify the relationship between in situ human chondrocyte morphology, phenotype, and matrix metabolism.

## References

[1] Buckwalter JA, Mankin HJ, Grodzinsky AJ (2005). Articular cartilage and osteoarthritis. Instr Course Lect.

[2] Goldring MB, Goldring SR (2007). Osteoarthritis. J Cell Physiol.

[3] Aigner T, Soder S, Gebhard PM (2007). Mechanisms of disease: role of chondrocytes in the pathogenesis of osteoarthritis-structure, chaos and senescence. Nat Clin Pract Rheumatol.

[4] Squires GR, Okouneff S, Ionescu M (2003). The pathobiology of focal development in aging human articular cartilage and molecular matrix changes characteristic of osteoarthritis. Arthritis Rheum.

[5] Bush PG, Hall AC (2003). The volume and morphology of chondrocytes within non-degenerate and degenerate human articular cartilage. Osteoarthritis Cartilage.

[6] Bush PG, Wokosin DL, Hall AC (2007). Two-versus one photon excitation laser scanning microscopy: critical importance of excitation wavelength. Front Biosci.

[7] Kouri JB, Arguello C, Luna J (1998). Use of microscopical techniques in the study of human chondrocytes from osteoarthritic cartilage: an overview. Microsc Res Tech.

[8] Holloway I, Kayser M, Lee DA (2004). Increased presence of cells with multiple elongated processes in osteoarthritic femoral head cartilage. Osteoarthritis Cartilage.

[9] Tesche F, Miosge M (2005). New aspects of the pathogenesis of osteoarthritis: the role of fibroblast-like chondrocytes in late stages of the disease. Histol Histopathol.

[10] McGlashan SR, Cluett EC, Jensen CG (2008). Primary cilia in osteoarthritic chondrocytes: from chondrons to clusters. Dev Dyn.

[11] Cancedda R, Descalzi-Cancedda F, Castagnola P (1995). Chondrocyte differentiation. Int Rev Cytol.

[12] von der Mark K, Gauss V, von der Mark H (1977). Relationship between cell shape and type of collagen synthesised as chondrocytes lose their cartilage phenotype in culture. Nature.

[13] Buschmann MD, Gluzband YA, Grodzinsky AJ (1992). Chondrocytes in agarose culture synthesize a mechanically functional extracellular matrix. J Orthop Res.

[14] Bonaventure J, Kadhom N, Cohen-Solal L (1994). Re-expression of cartilage-specific genes by dedifferentiated human articular chondrocytes cultured in alginate beads. Exp Cell Res.

[15] Moos V, Fickert S, Muller B (1999). Immunohistological analysis of cytokine expression in human osteoarthritic and healthy cartilage. J Rheumatol.

[16] Tetlow LC, Adlam DJ, Woolley DE (2001). Matrix metalloproteinase and proinflammatory cytokine production by chondrocytes of human osteoarthritic cartilage. Arthritis Rheum.

[17] Westacott CI, Whicher JT, Barnes IC (1990). Synovial fluid concentration of five different cytokines in rheumatic diseases. Ann Rheum Dis.

[18] Marini S, Fasciglione GF, Monteleone G (2003). Correlation between cartilage degradation observed by arthroscopy and synovial proteinases activities. Clin Biol.

[19] Goldring MB, Birkhead J, Sandell LJ (1988). Interleukin 1 suppresses expression of cartilage-specific types II and IX collagens and increases types I and III collagens in human chondrocytes. J Clin Invest.

[20] Tyler JA, Saklatvala J (1985). Pig IL-1 (catabolin) induces resorption of cartilage proteoglycan and prevents synthesis of proteoglycan and collagen. Br J Rheum.

[21] Poole A (1997). Articular cartilage chondrons: form, function and failure. J Anat.

[22] Guilak F, Alexopoulos LG, Upton ML (2006). The pericellular matrix as a transducer of biomechanical and biochemical signals in articular cartilage. Ann N Y Acad Sci.

[23] Alexopoulos LG, Setton LA, Guilak F (2005). The biomechanical role of the chondrocyte pericellular matrix in articular cartilage. Acta Biomater.

[24] Soder S, Hambach L, Lissner R (2002). Ultrastructural localisation of type VI collagen in normal adult and osteoarthritic human articular cartilage. Osteoarthritis Cartilage.

[25] Pullig O, Weseloh G, Swoboda B (1999). Expression of type VI collagen in normal and osteoarthritic human cartilage. Osteoarthritis Cartilage.

[26] Hambach L, Neureiter D, Zeiler G (1998). Severe disturbance of the distribution and expression of type VI collagen chains in osteoarthritic articular cartilage. Arthritis Rheum.

[27] Jones CW, Smolinski D, Keogh A (2005). Confocal laser scanning microscopy in orthopaedic research. Prog Histochem Cytochem.

[28] Amin AK, Bush PG, Huntley JS (2008). Osmolarity influences chondrocyte death in wounded articular cartilage. J Bone Joint Surg Am.

[29] Hunziker EB, Peyron JG Articular cartilage structure in humans and experimental animals. Articular cartilage and osteoarthritis.

[30] Dudhia J (2005). Aggrecan aging and assembly in articular cartilage. Cell Mol Life Sci.

[31] McCollum R, Martel-Pelletier J, DiBattista J (1991). Regulation of interleukin 1 receptor in human articular chondrocytes. J Rheumatol.

[32] Fan Z, Soder S, Oehler S (2007). Activation of interleukin-1 signalling cascades in normal and osteoarthritic articular cartilage. Am J Pathol.

[33] Kobayashi M, Squires GR, Mousa A (2005). Role of interleukin-1 and tumor necrosis factor α in matrix degradation of human osteoarthritic cartilage. Arthritis Rheum.

[34] Plaas A, Osborn B, Yoshihara Y (2007). Aggrecanolysis in human osteoarthritis: confocal localization and biochemical characterisation of ADAMTS5-hyaluronan complexes in articular cartilage. Osteoarthritis Cartilage.

[35] Page-McCaw A, Ewald AJ, Werb Z (2007). Matrix metalloproteinases and the regulation of tissue remodelling. Nat Rev Mol Cell Biol.

[36] Loeser R (1997). Growth factor regulation of chondrocyte integrins. Arthritis Rheum.

[37] Ross JM, Sherwin AF, Poole CA (2006). In vitro culture of enzymatically isolated chondrons: a possible model for the initiation of osteoarthritis. J Anat.

[38] Fukui N, Zhu Y, Maloney WJ (2003). Stimulation of BMP-2 expression by pro-inflammatory cytokines IL-1 and TNF-α in normal and osteoarthritic chondrocytes. J Bone Joint Surg Am.

[39] Alexopoulos LG, Youn I (2009). Developmental and osteoarthritic changes to *Col6a1*-knockout mice. Arthritis Rheum.

